# Inside the bulk of magnetic nanocomposites: I am not what I am^1^

**DOI:** 10.1107/S160057671502066X

**Published:** 2015-11-28

**Authors:** Heinrich B. Stuhrmann

**Affiliations:** a IBS, Grenoble, France; b HZG, Geesthacht, Germany

**Keywords:** small-angle neutron scattering, correlation function, micromagnetics, magnetic materials

## Abstract

An unexpected and not always easily discernible feature in the picture of magnetic neutron scattering is widening the outlook on micromagnetic architecture [Mettus & Michels (2015). *J. Appl. Cryst.***48**, 1437–1450].

An unexpected and not always easily discernible feature in the picture of magnetic neutron scattering is widening the outlook on micromagnetic architecture (Mettus & Michels, 2015 ▸). It was first observed with magnetic nanocomposites.

Since their discovery in 1988 ▸ (Yoshizawa *et al.*, 1988 ▸), soft magnetic nanocomposites have attracted wide interest owing to their exceptional magnetic properties. Their high permeability, low hysteresis loss, large saturation and remnant magnetization, combined with a high Curie temperature, open important industrial applications. There is an increasing use of such materials as core elements in transformers, noise filters and antennae, and, more recently, in synchrotron RF systems, for instance.

Initially, the precursor of soft magnetic nanocomposites was a melt-spun Fe–Si–B alloy containing small amounts of Nb and Cu. The amorphous sample was annealed, so as to obtain an Fe–Si solid solution with particle sizes of about 10 nm. Since then, important improvements to the soft ferromagnetic properties have been achieved by tailoring the chemistry and optimizing the microstructure.

Progress in the field of nanomagnetism relies on the continuous development of observational microscopy techniques, such as spin-polarized scanning tunnelling microscopy and holography, or Kerr microscopy and magnetic X-ray diffraction. Neutron scattering is a unique tool in the study of magnetism, as it provides access to the structure and dynamics of magnetic materials on a wide range of length and time scales. As neutrons penetrate deeply into matter, they are ideally suited for the study of bulk properties.

It was about ten years ago when, quite unexpectedly, a clover-leaf scattering intensity distribution from a soft magnetic iron-based alloy of the Nanoperm family (Fe_89_Zr_7_B_3_Cu) was observed, which was attributed at the time to dipolar stray fields around the nanosized iron particles embedded in an amorphous magnetic matrix of lesser saturation magnetization (Michels *et al.*, 2006 ▸). From the dependence of the intensity on the applied magnetic field and momentum transfer, it was concluded that the dipole field induces nanoscale spin disorder, correlating spin misalignment of neighbouring particles and the matrix over several particle spacings (Fig. 1 ▸).

The article by Mettus & Michels (2015 ▸) (of the Physics and Materials Science Research Unit, University of Luxembourg) reports on correlation functions of bulk magnetic materials obtained from magnetic small-angle neutron scattering (magnetic SANS). An important impact for this study came from magnetic SANS studies of nanocomposites, where spin misalignment had been postulated. Clearly, the standard model of magnetic SANS – relying on homogenously magnetized domains – needed to be extended so as to include the features shown in Fig. 1 ▸. In other words, the classical particle–matrix concept is not adapted to the complex magnetic textures of nanocomposites or, more generally speaking, to that of bulk magnetic materials. In fact, it is the continuum theory of micromagnetics which provides an adequate framework for computing the equilibrium magnetization distribution of such materials and, hence, the magnetic SANS cross section. The special feature of the paper by Mettus & Michels is the projection of micromagnetic theory into real space by calculating the correlation function of the spin-misalignment SANS cross section.

The description of the magnetization in mesomorphous structures is an inherently difficult (nonlinear) task. Some of its problems have been avoided by focusing on small spin misalignment, *i.e.* the transverse spin components are assumed small compared with the longitudinal one in the direction of the external magnetic field. In the high-field limit, the linearized balance-of-torques equation then provides expressions for the Fourier components of the magnetization along the Cartesian coordinates which enter into the cross sections of magnetic scattering (Michels, 2014 ▸).

As spin-misalignment scattering can be observed at any polarization of the neutron beam, the discussion is restricted to unpolarized neutron scattering. The scattering cross section then consists of the ‘classical’ nuclear and magnetic SANS cross section, measured at complete saturation and characterized *e.g.* by an anisotropic sin^2^θ-type scattering intensity distribution, and the spin-misalignment SANS cross section, where the transverse spin components together with the longitudinal component give rise to a wealth of angular anisotropies, ranging from spike-type to elliptical to clover-leaf-shaped patterns (compare Figs. 1 ▸ *a*–1 ▸ *d*). Spin-misalignment SANS contains a wealth of information on the bulk magnetization. The spin-misalignment correlation length is deduced from the profile of the correlation function (Figs. 1 ▸ *e*–1 ▸ *h*), and the dependence of this correlation length on the internal field is mediated by the exchange-stiffness constant. Another decisive parameter in the theory is the ratio between the fluctuations in the anisotropy field and the jump of the magnetostatic field at internal interfaces (*e.g.* grain boundaries), which is determined experimentally from the deconvolution of the spin-misalignment scattering cross section into its anisotropy field related and magnetostatic parts.

While the authors discuss in great detail some unusual properties of the correlation function due to spin misalignment, like its deviation from Porod’s law, the essential message of the paper is undoubtedly that of an extension of magnetic SANS by a new dimension: modest though the new feature of magnetic neutron scattering appears to be, its impact on the micromagnetic description of SANS might be considerable. We remind the reader that the anomalous form factors in X-ray scattering evolved from small but necessary corrections to become a cornerstone of synchrotron X-ray diffraction. Spin-misalignment scattering might well take the same path in neutron scattering.

## Figures and Tables

**Figure 1:**
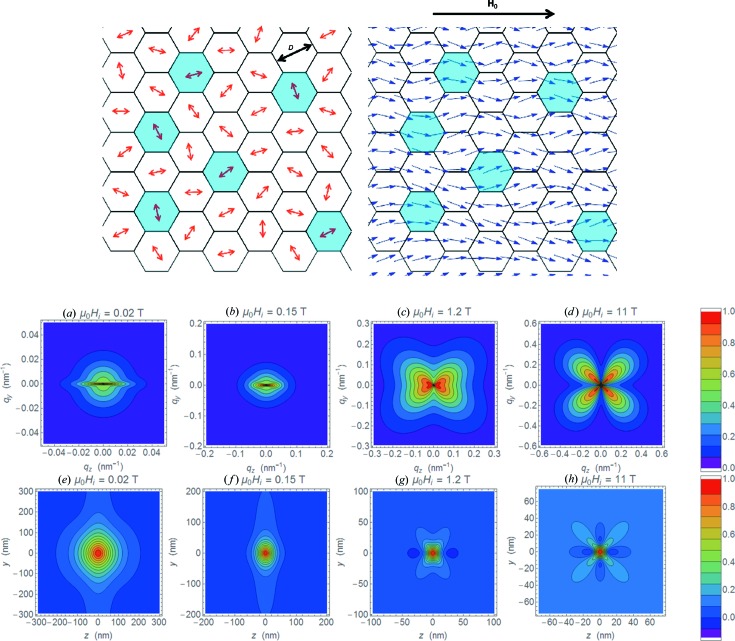
(Top) The model of magnetic microstructure and the comparison of its scattering pattern with correlation functions. (Top left) A sketch of an idealized two-dimensional grain microstructure. Regions with different materials parameters (*e.g.* saturation magnetization) are distinguished by their colour. (Top right) The smooth variation of the magnetization near saturation in the presence of an external field **H**_0_ is characterized by a correlation length which extends over several cells. (Bottom) (*a*)–(*d*) Contour plots of normalized spin-misalignment SANS cross section at applied magnetic fields as indicated. (*e*)–(*h*) The corresponding two-dimensional correlation functions. Courtesy of Mettus & Michels (2015 ▸).
